# Effectiveness and Safety of a Supplement Containing a Pharmacologically Active Basidiomycete Mushroom for Chronic Fatigue and Post–COVID-19 Fatigue Syndrome: Protocol for a Randomized Controlled Trial

**DOI:** 10.2196/82633

**Published:** 2026-01-20

**Authors:** Yunqing Xun, Tung Leong Fong, Guang Chen, Yibin Feng, Linda Chan, Ning Wang

**Affiliations:** 1 School of Chinese Medicine Li Ka Shing Faculty of Medicine The University of Hong Kong Hong Kong China (Hong Kong); 2 Bau Institute of Medical and Health Sciences Education Li Ka Shing Faculty of Medicine The University of Hong Kong Hong Kong China (Hong Kong); 3 Department of Family Medicine and Primary Care The University of Hong Kong-Shenzhen Hospital Hong Kong China (Hong Kong); 4 School of Clinical Medicine Li Ka Shing Faculty of Medicine The University of Hong Kong Hong Kong China (Hong Kong)

**Keywords:** CP003, chronic fatigue, post–COVID-19 fatigue syndrome, randomized controlled trial, protocol

## Abstract

**Background:**

Chronic fatigue syndrome, or myalgic encephalomyelitis, is characterized by persistent, unexplained exhaustion unalleviated by rest, with a pathophysiology distinct from underlying medical conditions. It is diagnostically complex due to symptoms that overlap with other disorders and the absence of definitive biomarkers, contributing to limited therapeutic options in current medicine. *Lingzhi*, a pharmacologically active basidiomycete mushroom, has been empirically used in traditional Chinese medicine for two millennia. This study has a dual focus: (1) systematically evaluating the efficacy and safety of lingzhi in managing chronic fatigue and post–COVID-19 fatigue syndrome and (2) elucidating its clinical associations with inflammatory, immune, and oxidative stress biomarkers to uncover potential therapeutic mechanisms.

**Objective:**

The primary objective is to investigate whether a 6-week intake of CP003, a lingzhi-containing supplement, can reduce physical and mental fatigue in patients with chronic fatigue syndrome (ie, myalgic encephalomyelitis) or post–COVID-19 fatigue. Secondary objectives include assessing its effects on sleep quality, anxiety, depression, and general health status and exploring associations with inflammatory, immune, and oxidative stress biomarkers.

**Methods:**

This randomized, waitlist-controlled trial will enroll 130 participants in Hong Kong, equally allocated (1:1) to either the CP003 intervention group or a waitlist control group. The intervention period spans 6 weeks, followed by a 6-week follow-up phase to assess the sustained effects. The trial data will be managed using REDCap (Research Electronic Data Capture) and analyzed via an intention-to-treat approach with inverse probability weighting for missing data, assessed through generalized linear regression (adjusted for covariates and interaction terms) at 6 and 12 weeks and supplemented by subgroup and sensitivity analyses in R, with results reported as mean differences (with 95% CIs) and *P*<.05 considered significant.

**Results:**

This study was funded in March 2024 (ITF/PRP/029/24FX). As of December 2025, all participants had been enrolled, but data entry had not yet commenced. Data analysis will commence after the completion of the 12-week follow-up for all participants. Results are expected to be submitted for publication in mid-2026. Safety outcomes will also be assessed and reported.

**Conclusions:**

This trial will evaluate the efficacy and safety of CP003 for chronic fatigue and post–COVID-19 fatigue syndrome and explore potential underlying mechanisms of action. The findings will provide experimental data to inform future clinical applications and research on traditional Chinese medicine–based interventions for fatigue-related disorders.

**Trial Registration:**

ClinicalTrials.gov NCT06739720; https://clinicaltrials.gov/study/NCT06739720

**International Registered Report Identifier (IRRID):**

PRR1-10.2196/82633

## Introduction

### Background and Rationale

Chronic fatigue is a term that is often used interchangeably with chronic fatigue syndrome (CFS), also known as myalgic encephalomyelitis (ME), which is a complex, long-term medical disorder characterized by extreme fatigue that cannot be explained by any underlying medical condition. The fatigue is not alleviated by rest and may worsen with physical or mental activity [1]. In addition to fatigue, people with CFS/ME may experience other symptoms, such as muscle pain, joint pain, sleep disturbances, headaches, cognitive difficulties, and postexertional malaise. Estimates of the prevalence of CFS/ME vary widely, ranging from 0.2% to 2.6% of the population depending on the diagnostic criteria used and the population studied. Some studies suggest that approximately 0.4% to 1% of the population may be affected by CFS/ME. CFS/ME can affect people of any age, but it is most commonly diagnosed in people aged between 40 and 60 years, with a female-to-male ratio ranging from 2:1 to 4:1 [2]. Diagnosing CFS/ME can be challenging due to the lack of a specific diagnostic test and the similarity of its symptoms to many other medical conditions. Due to the complex nature and the challenges in diagnosing and studying the condition, there is currently no cure for CFS/ME, but several interventions can help manage symptoms, improve the quality of life, and support daily functioning—including pacing and energy management, cognitive behavioral therapy, graded exercise therapy, sleep management, and symptom medications [3]. The treatment approach is often individualized, as the severity and types of symptoms can vary greatly among patients.

The exact pathological mechanism of CFS is not yet fully understood, but several theories have been proposed. It is likely that a combination of factors contributes to the development and persistence of the condition. Studies have suggested that CFS may be associated with an abnormal immune response, possibly due to a viral infection [4]. People with CFS often have elevated levels of immune system molecules called cytokines that could contribute to the fatigue and influenza-like symptoms. Patients with CFS/ME may have higher levels of inflammatory markers, such as cytokines, or evidence of impaired immune function, such as decreased natural killer cell activity [5]. Oxidative stress is another possible mechanism involved in CFS/ME, as some studies have found that patients with CFS/ME may have reduced levels of antioxidants or impaired antioxidant defenses, which could make them more susceptible to oxidative stress [6]. Increased oxidative stress may lead to a decrease in the production of adenosine triphosphate, the primary energy source for cells. This reduced energy production may contribute to the fatigue experienced by patients with CFS/ME. Oxidative stress can cause damage to various cellular components, including lipids, proteins, and DNA. This damage may impair cellular function and contribute to the symptoms of CFS/ME [7]. Poor sleep quality can exacerbate CFS/ME and other symptoms associated with the condition. One of the key diagnostic criteria for CFS/ME is unrefreshing sleep, where patients do not feel rested or refreshed even after a full night’s sleep. This can be due to poor sleep quality, difficulty falling asleep, or frequent awakenings during the night, all of which are characteristic of insomnia [8]. People with CFS/ME often experience various sleep disturbances, such as sleep onset insomnia (difficulty falling asleep), sleep maintenance insomnia (difficulty staying asleep), and early morning awakenings. These sleep disturbances can contribute to the fatigue and cognitive difficulties experienced by patients with CFS/ME. They may also have disruptions in their circadian rhythm or sleep-wake cycle that can lead to insomnia or other sleep disorders. This disruption can result from a combination of factors, including hormonal imbalances, immune system dysfunction, and external factors, such as light exposure or lifestyle habits [9]. Specifically, there is growing evidence that some individuals recovering from COVID-19 may develop symptoms similar to those of CFS/ME, suggesting a potential link between chronic fatigue and post–COVID-19 fatigue syndrome. Although CFS and post–COVID-19 fatigue are heterogeneous in etiology, they exhibit several overlapping biological mechanisms. For example, both CFS/ME and post–COVID-19 fatigue are associated with persistent immune activation and elevated levels of proinflammatory cytokines and autoantibodies. Some studies also reported a downregulation of activities in hypothalamic pituitary axes in patients with CFS/ME and post–COVID-19 fatigue. These mechanisms can contribute to prolonged fatigue and other systemic symptoms. Both CFS/ME and post–COVID-19 syndrome are characterized by persistent fatigue, unrefreshing sleep, cognitive difficulties, and other symptoms that can significantly impact daily functioning. In the meantime, fatigue is a common and significant symptom reported by many individuals experiencing post–COVID-19 syndrome. Understanding the clinically relevant mechanism of CFS/ME will be useful for the development of effective management of CFS/ME [10].

*Lingzhi*, also known as “*reishi* mushroom” or *Ganoderma lucidum*, is a type of medicinal mushroom that has been used in traditional Chinese medicine (TCM) and other East Asian medicinal practices for more than 2000 years. It is a woody, shelf-like mushroom that is typically reddish-brown in color with a glossy, varnished appearance. Lingzhi is believed to have numerous medicinal properties and health benefits. It has been used as an herbal remedy to promote health, longevity, and overall well-being. Some potential benefits of lingzhi include boosting the immune system, reducing stress and anxiety, improving liver function, supporting heart health, and providing antiaging effects. Numerous contemporary comprehensive research efforts have conclusively demonstrated the promising medical capabilities of *G lucidum*, a medicinal mushroom, in various areas, such as combating cancer, safeguarding liver health, reducing inflammation, regulating immune system functions, neutralizing harmful free radicals, and providing protection against viral infections. Clinical studies observed that consumption of *G lucidum* does not lead to the disruption of blood clotting processes and the immune system in healthy individuals, indicating its safety and tolerability in human use [11,12]. In patients with lower urinary tract symptoms, the use of lingzhi was well tolerated and provided an improvement in the International Prostate Symptom Score [13,14]. Lingzhi can significantly improve antioxidant enzymes [15], enhance physical fitness [16], and maintain the immune functions of patients who are immunocompromised [17]. However, the effect of lingzhi on diabetes remains controversial and unconfirmed [18,19]. The traditional use of lingzhi and the results of recent clinical trials support its potential application in the management of CFS in patients with or without a history of COVID-19 infections.

Randomized controlled trials are required to determine whether CP003, a lingzhi-containing supplement, is effective and safe for chronic fatigue and post–COVID-19 fatigue syndrome. Because there is no evidence-based pharmacological treatment for these fatigue conditions, the ideal comparator group is a placebo. Considering the feasibility of this trial, we designed a randomized, waitlist-controlled trial for this study.

### Objectives

The primary objective of this study is to investigate whether the intake of lingzhi-containing CP003 over 6 weeks can reduce physical and mental fatigue in patients with both CFS/ME and post–COVID-19 fatigue syndrome. We will primarily focus on the effectiveness and safety of CP003-containing lingzhi for addressing chronic fatigue and post–COVID-19 fatigue symptoms as well as its clinical associations with various factors, including inflammation, immunity, and oxidative aging.

## Methods

### Trial Design

This is a randomized, waitlist-controlled trial on patients stratified by CFS/ME or post–COVID-19 fatigue syndrome who will receive the intervention for 6 weeks, followed by a 6-week follow-up. Because there is no approved pharmaceutical intervention for CFS/ME or post–COVID-19 fatigue, a waitlist control without a pharmacologically active drug is designed in this trial. The study is registered at the ClinicalTrials.gov (NCT06739720).

**Figure 1:**
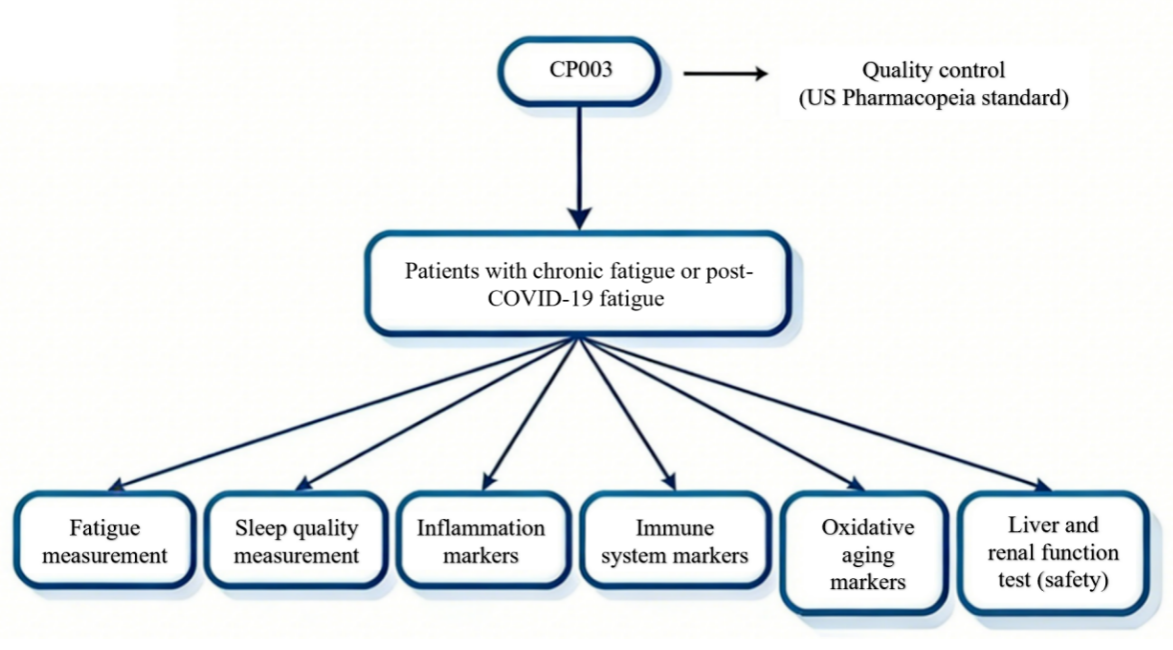
Study flowchart for this randomized, waitlist-controlled trial evaluating CP003 in participants with chronic fatigue syndrome or myalgic encephalomyelitis (CFS/ME) or post–COVID-19 fatigue in Hong Kong.

This study will be conducted in accordance with the ethical principles of the Declaration of Helsinki. The representative work flowchart is shown in Figure 1. This protocol is reported in accordance with the SPIRIT (Standard Protocol Items: Recommendations for Interventional Trials) 2013 statement.

### Ethical Considerations

#### Overview

This trial has been approved by the University of Hong Kong/Hospital Authority Hong Kong West Cluster Institutional Review Board (UW 24-639). All procedures will be conducted in accordance with the ethical principles of the Declaration of Helsinki.

#### Informed Consent

Written informed consent will be obtained from all participants before any study-related procedures. Research staff will explain the study’s purpose, procedures, potential risks and benefits, and the right to withdraw at any time without penalty, using a detailed information sheet. Consent will be obtained electronically via the secure REDCap (Research Electronic Data Capture; Vanderbilt University) platform. Participants will have the opportunity to discuss any questions with a member of the research team before providing consent. The consent process and documentation have been approved by the aforementioned institutional review board.

#### Participation and Compensation

Participation in this project is entirely voluntary, and patients are not charged for anything associated with it. To assist with patients’ travel to the Specialist Clinical Centre for Teaching and Research, and the blood sampling center, they will be reimbursed 800 HKD (US $104) to cover travel expenses. This reimbursement will be provided after the completion of the 12-week follow-up and is intended purely to compensate for travel costs incurred by participating in the study. Patients’ participation or decision to withdraw will not affect their access to medical care in this center.

#### Confidentiality and Privacy

The privacy rights of all participants will be strictly upheld. Personally identifiable information will be collected only when absolutely necessary to conduct the study. All data will be deidentified before analysis, with participant codes replacing any direct identifiers, such as names, initials, hospital numbers, or specific dates. In any publications or presentations, no identifiable details will be disclosed. Should the publication of any potentially identifiable information in case descriptions, images, or other materials be deemed scientifically essential, separate and explicit written consent for publication will be obtained from the affected individuals, and the manuscript will be presented to them for review before submission.

### Study Setting

This study will take place at the Specialist Chinese Medicine Clinic, School of Chinese Medicine, The University of Hong Kong, Hong Kong.

### Patient and Public Involvement

Patients were not involved in the design or development of this study protocol. Participants meeting the eligibility criteria (ie, aged 40-60 years, diagnosed with CFS/ME per the Fukuda criteria or with post–COVID-19 persistent fatigue, and able to complete the 6-week intervention period and online fatigue assessments) were recruited through advertisement campaigns and outpatient clinics in Hong Kong. Potential participants were provided with detailed study information, including intervention requirements and assessment procedures, before obtaining written informed consent. Exclusion criteria, such as recent use of supplements or herbal medicine, acute inflammation, pregnancy, or severe comorbidities, were clearly communicated during recruitment to ensure appropriate enrollment. Although study results will not be disseminated directly to participants, individuals may request outcome summaries or the published manuscript upon trial completion.

### Eligibility Criteria

#### Inclusion Criteria

Participants are included in this trial if they meet the following criteria: aged between 40 and 60 years, diagnosed with CFS/ME according to the Fukuda criteria or experiencing persistent fatigue following post–COVID-19 condition [20], capable of undergoing a 6-week intervention program, and able to complete online validated fatigue assessments. Recruitment will be conducted through both advertisement campaigns and outpatient clinic enrollments in Hong Kong, targeting individuals with CFS/ME or post–COVID-19 condition who meet the eligibility requirements.

#### Exclusion Criteria

The exclusion criteria are as follows: (1) patients who have taken any type of supplements or herbal medicine within the past 6 months, (2) patients who have serious medical conditions that might limit their participation in this intervention, (3) patients who are diagnosed with acute inflammation, or (4) patients who are pregnant or planning to become pregnant in the next 3 months.

### Randomization and Blinding

After screening and baseline assessments, eligible participants will be randomly assigned to the CP003 intervention group or waitlist control group in a 1:1 ratio stratified by CFS/ME and post–COVID-19 condition. The CP003 intervention group received a therapeutic dose (5 capsules taken orally once per day for 6 weeks). The waitlist control group will wait for 6 weeks after the randomization and then start to receive CP003 capsules (5 capsules taken orally once per day for 6 weeks), followed by a 6-week follow-up. The random sequences generated in the R statistical package will have a block of 8 in each cycle, consisting of 2 CFS/ME control groups, 2 post–COVID-19 condition control groups, 2 CFS/ME intervention groups, and 2 post–COVID-19 condition intervention groups. Assignments were concealed in sealed opaque envelopes and were only opened after eligibility was confirmed and consent was obtained. The research study staff who recruit and screen the patients and the assessors who conduct baseline and follow-up assessments will be blinded to treatment allocation.

### Interventions

Participants assigned to the intervention group will receive 5 CP003 capsules daily for 6 weeks, followed by a 6-week follow-up. CP003 is a Chinese medicine nutritional supplement used in Hong Kong, manufactured by Chinese Pharmaceuticals Co Ltd. Per the manufacturer’s guidelines, a complete treatment course consists of 6 weeks of CP003. Quality control will ensure that the similarity of multiple batches exceeds 96%, as tested by ultraperformance liquid chromatography with fingerprint analysis. During the intervention period, patients will be asked to complete a supplement intake diary to record any missed doses and any additional supplements taken. At the 6-week visit, participants are required to return any unused trial capsules.

**Figure 2:**
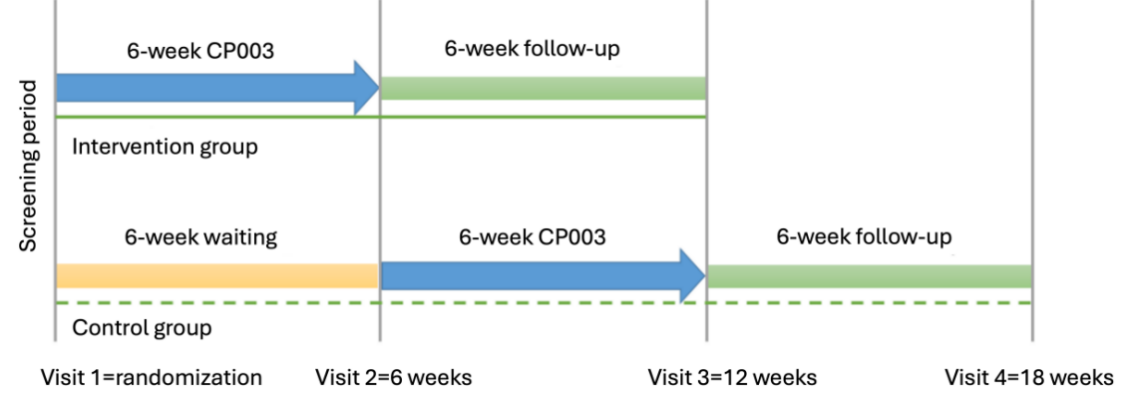
Schematic overview of the trial design and participant timeline for the CP003 intervention study. Patients with fatigue will be randomly assigned (1:1) to the intervention group and control group.

The control group (ie, the waitlist) will wait for 6 weeks after randomization before receiving CP003 capsules and then start taking 5 capsules daily for 6 weeks, followed by a 6-week follow-up. During the waiting period, participants will be asked to complete a supplement intake diary to record any supplements they take in their daily lives. The overview of the study design is presented in Figure 2.

### Outcomes

Outcome measures, including the Chalder Fatigue Scale score (Likert score 0-4 for 11 items, with higher scores indicating greater fatigue) [21], Fatigue Severity Score (Likert score 1-7 for 9 items, where higher scores indicate more severe fatigue) [22], Patient-Reported Outcomes Measurement Information System (PROMIS) Short Form Fatigue 7a survey (a 5-point Likert scale of 7 items, with higher scores indicating greater fatigue) [23], 36-Item Short Form Health Survey (SF-36; ranging 0-100, with higher scores indicating better health status) [23], Hospital Anxiety and Depression Scale (HADS; ranging 0-21, with higher scores indicating more severe symptoms) [24], and Pittsburgh Sleep Quality Index (ranging 0-21, with higher scores indicating worse sleep quality) [25]. Additional measures include an outcome expectations scale; physical examinations (assessing tongue and pulse, blood pressure, and body temperature); serum markers for inflammation, immunity, and oxidative aging; as well as laboratory tests for safety assessments, including complete blood count, renal function (blood urea nitrogen and creatinine), and liver function (aspartate aminotransferase and alanine aminotransferase).

### Primary Outcome Measures

The primary outcome is the mean difference in the Chalder Fatigue Scale score from baseline to week 6.

### Secondary Outcome Measures

The secondary outcomes include the mean difference in the Chalder Fatigue Scale scores from baseline to 12 weeks, the mean difference in the SF-36 scores from baseline to both 6 and 12 weeks, the mean difference in the Fatigue Severity Scores from baseline to both 6 and 12 weeks, the mean difference in the PROMIS Short Form Fatigue 7a survey scores from baseline to both 6 and 12 weeks, the mean difference in the HADS scores from baseline to both 6 and 12 weeks, and the mean difference in the Pittsburgh Sleep Quality Index scores from baseline to both 6 and 12 weeks.

### Blood Tests

Patients will be asked to undergo a blood test at baseline and at the end of the intervention program to investigate the inflammation-, immune-, and oxidative aging–related measures. For chronic inflammation and immunity assessment, the BD Cytometric Bead Array system will be used to quantify the level of inflammation panel (interleukin-10, interleukin-12 p70, interleukin-1β, and interleukin-6), T helper 1 cells and T helper 2 cells panel (interleukin-2, interleukin-4, interleukin-5, tumor necrosis factor, and IFN-γ), B cell activation panel (CD79b, B cell linker protein, Btk, Syk, and PLCγ), T cell activation panel (TCRz, SLP-76, ZAP-70, Pyk2, Itk), and the complements panel (C4a, C3a, and C5a). The plasma level of C-reactive protein will be determined by the enzyme-linked immunosorbent assay. The oxidative aging maker in the plasma, including antioxidative enzyme activities of superoxide dismutase, glutathione peroxidase, and catalase as well as GSH (reduced form of glutathione), GSSG (oxidized form of glutathione), and malondialdehyde concentration, will be determined by colorimetric biochemical assays. For cardio-related markers, triglyceride, low-density lipoprotein cholesterol, high-density lipoprotein cholesterol, and total cholesterol will be tested. Fasting plasma glucose will also be evaluated. Blood vessel elasticity will be evaluated by the arterial velocity-pulse index and arterial pressure-volume index by a noninvasive PASESA detector (Win Horizon).

### Sample Size

The sample size for this study is based on the minimal important difference threshold of 2 points, which is the expected mean difference between groups [26]. Using a 2-sided hypothesis test at a significance level of .05 with an allocation ratio of 1:1, a sample size of 110 is calculated to achieve approximately 80% power to detect this effect size difference. Considering a 15% loss to follow-up or dropout rate, the final estimated sample size for this trial is 130.

### Feasibility and Process Evaluation

To assess the feasibility of the trial and the acceptability of the CP003 intervention, we will monitor the following process measures: (1) recruitment rate (ie, the number of participants enrolled per month), (2) retention and dropout rates at each study visit, (3) intervention adherence calculated based on the count of the remaining capsules and self-reported intake diaries, and (4) participant satisfaction with the intervention and study procedures assessed via a brief survey at the final visit. These measures will inform the practicality of conducting a larger definitive trial.

### Recruitment

Patients in this trial will be recruited through both advertisements and outpatient clinic enrollments in Hong Kong.

### Adverse Events

Adverse events will be assessed using the Treatment Emergent Symptom Scale and evaluated during the whole process as well as laboratory tests (eg, whole blood count, renal, and liver function tests), if needed. All clinical adverse events will be recorded in terms of intensity (mild, moderate, or severe), duration, outcome, and relationship to the study.

### Handling and Storage of Personal Data and Study Data

Data will be managed by and accessible only to authorized investigators and designated personnel on a password-protected system throughout the study period. In addition, an independent data and safety monitoring committee, separate from sponsors, researchers, and stakeholders, will convene quarterly to oversee the safety and effectiveness of the research in the medium term. The principal investigator will have access to the personal and study data during and after the study.

Our project team will ensure the security of raw data by storing it in a locked cabinet within a secure room with limited access. Data entry will be performed on a password-protected computer accessible solely to team members. Throughout and following the study, only designated team members will have the authority to access raw data or study records. Upon study completion, raw data will be preserved for a period of 5 years and subsequently disposed of, unless an application for extended retention is sanctioned by the institutional review board. Before statistical analysis, any personal identifying information will be expunged from the dataset.

### Data Processing and Analysis

The primary analysis will follow the intention-to-treat principle. The between-group difference in the primary outcome, defined as the mean change in the Chalder Fatigue Scale score from baseline to week 6, will be assessed using a generalized linear regression model, adjusted for the baseline score and stratification factors of CFS/ME versus post–COVID-19 fatigue. Results will be presented as the adjusted mean difference with a 95% CI. For participants lost to follow-up, inverse probability weighting will be used to address potential bias from missing data. Analysis of secondary outcomes and biomarker data will use similar generalized linear models at 6 and 12 weeks, adjusted for prespecified covariates, including time since COVID-19 infection, vaccination doses, routine exercise pattern, and baseline sleep quality. Subgroup analyses will be conducted based on gender, age category, and history of COVID-19 infection. Sensitivity analyses, including those using multiple imputation for missing data, will be performed. All analyses will be conducted using R software (version 4.5.2; R Foundation for Statistical Computing), with a 2-sided *P* value of <.05 considered statistically significant.

### Withdrawal and Dropout

If participants use a combination of treatments that are prohibited by the study protocol, withdraw their consent, or cease communication, they will be excluded from the study. Researchers will report the reasons for withdrawal and dropout and acquire the time of last treatment and outcome measures where possible.

### Consent or Assent

Research staff will ask interested participants to provide consent via the secure REDCap electronic consent platform. Participants will receive information regarding the trial electronically and have the opportunity to discuss the trial specifics and meet with a research team member, virtually or in person, before deciding on participation.

### Dissemination Policy

Upon study completion, the main findings will be disseminated to patients, primary care providers, and physicians through the hospital authority network in Hong Kong. Results will be presented at academic conferences and published in peer-reviewed journals.

## Results

This is a study protocol, and no results from the trial are available at this stage. This study received funding in March 2024 (grant ITF/PRP/029/24FX). Participant recruitment concluded in September 2025. Follow-up for each patient will be 6 weeks. The total duration of the study will be approximately 24 months. As of December 2025, all participants had been enrolled, but data entry had not yet commenced. Data collection, including baseline and all follow-up assessments, will proceed according to the timeline presented in Figure 2. Data analysis will commence after all participants complete the 12-week follow-up. Results are expected to be submitted for publication in mid-2026. Upon trial completion, we will report the between-group differences in primary and secondary outcomes, such as the Chalder Fatigue Scale, Fatigue Severity Score, SF-36, PROMIS fatigue 7a, HADS, and the Pittsburgh Sleep Quality Index, at the 6- and 12-week time points. Changes in inflammatory, immune, and oxidative stress biomarkers from baseline to the end of intervention will also be presented. Safety outcomes will be assessed through adverse event monitoring, and laboratory tests will be reported.

In case of any changes to the protocol, the investigators, clinical research assistants, the ethics committee, and the sponsor will be immediately informed via email.

## Discussion

Our protocol uses a randomized, waitlist-controlled trial design to achieve two primary objectives: (1) systematically evaluate the efficacy and safety of CP003 for CFS/ME and post–COVID-19 fatigue management and (2) elucidate clinical correlations between CP003 and key biomarkers of inflammation, immune function, and oxidative stress to reveal potential therapeutic mechanisms. This design was selected based on 3 key considerations. First, the waitlist-controlled approach balances scientific rigor with ethical considerations by ensuring that all participants eventually receive the intervention while avoiding placebo-related ethical concerns in this vulnerable population [27]. Second, our dual clinical-biomarker assessment strategy overcomes limitations of purely subjective measures by combining validated questionnaires (ie, the Chalder Fatigue Scale) with quantitative biomarker analysis (eg, interleukin-6 and superoxide dismutase), as recommended by the International Consensus Criteria [28]. Third, our sample size (N=130) was powered to detect clinically meaningful 20% fatigue score reductions (α=.05 and power=80%) while accounting for 15% attrition, ensuring both statistical robustness and practical feasibility [29].

However, this study is subject to several limitations. The heterogeneity inherent in fatigue-related disorders, particularly the symptomatic overlap with conditions such as depression and fibromyalgia, may influence treatment response and generalizability. Although standardized fatigue scales were used, the primary efficacy evaluation remains dependent on patient-reported outcomes in the absence of definitive biomarkers. In addition, the 12-week study duration, comprising a 6-week intervention and 6-week follow-up periods, may be inadequate to fully assess the long-term benefits or potential risks associated with prolonged lingzhi administration. Future studies with extended observation periods and more rigorously stratified participant cohorts are warranted to corroborate these initial findings.

In conclusion, this trial adds to the expanding evidence base for natural interventions in fatigue-related conditions and provides new insights into the therapeutic potential of lingzhi as a TCM-derived agent. Should efficacy be established, subsequent research ought to prioritize several directions: (1) refining dosage and treatment protocols through dedicated dose-finding studies, (2) conducting comprehensive long-term safety evaluations in extended longitudinal settings, and (3) elucidating the underlying mechanisms in broader and more diverse patient populations. This study emphasizes the value of integrating traditional medicine with contemporary research frameworks to tackle complex, multifactorial syndromes, such as CFS/ME and post–COVID-19 fatigue syndrome, while underscoring the promising yet underexplored role of TCM-based formulations, such as lingzhi.

## Appendix

Multimedia Appendix 1 Peer-review report from The Innovation and Technology Commission Grant (Hong Kong).

## Abbreviations

CFS: chronic fatigue syndrome
HADS: Hospital Anxiety and Depression Scale
ME: myalgic encephalomyelitis
PROMIS: Patient-Reported Outcomes Measurement Information System
REDCap: Research Electronic Data Capture
SF-36: 36-Item Short Form Health Survey
TCM: traditional Chinese medicine

## Data Availability

The datasets generated or analyzed during this study are available from the corresponding author on reasonable request.
