# Supplementary material for: Effectiveness and Safety of a Supplement Containing a Pharmacologically Active Basidiomycete Mushroom for Chronic Fatigue and Post–COVID-19 Fatigue Syndrome: Protocol for a Randomized Controlled Trial
Source: JMIR Res Protoc. 2026 Jan 20;15:e82633. doi: 10.2196/82633 (PMC12869150; doi:10.2196/82633)
Supplement: Multimedia Appendix 1 [file resprot_v15i1e82633_app1.pdf]

original peer-review reports

The application has been reviewed by the TCM Expert advisor with the following comment(s):

1. Please clarify whether CTC is applied for CP003. Has it been registered in DH or listed in proprietary Chinese medicine registration in the Chinese Medicine Council of Hong Kong?

2. More detailed justification should be provided for the calculation of sample size, in particular evidence for 2 points with a standard deviation of 3.7.

3. Actually, two types of subjects will be recruited in the study: Chronic Fatigue Syndrome (CFS) and post-COVID fatigue. Although the two types share some similar pathophysiological and symptomatological characters, they are obviously heterogeneous in etiology. Please provide rationales/justification or make modification.

4. 6 weeks of treatment duration must be justified.

5. Due to the PI who is not qualified for clinically caring participants, how and who will be responsible for monitoring and taking care of participants' safety and adverse events.

6. Multiple assessments are proposed to measure outcomes, what is the primary and secondary outcomes. Who will be responsible for assessment. How assess.

7. There should be a member, such as general physician or family doctor to join the research team.
